# The Relationship of Nutritional Energy and Macronutrient Intake with Pregnancy Outcomes in Czech Pregnant Women

**DOI:** 10.3390/nu12041152

**Published:** 2020-04-20

**Authors:** Simona Najpaverova, Miroslav Kovarik, Marian Kacerovsky, Zdenek Zadak, Miloslav Hronek

**Affiliations:** 1 Department of Biological and Medical Sciences, Faculty of Pharmacy in Hradec Kralove, Charles University, 500 03 Hradec Kralove, Czech Republic; najpaves@faf.cuni.cz (S.N.); kovarikm@faf.cuni.cz (M.K.); 2 Department of Obstetrics and Gynecology, University Hospital Hradec Kralove, 500 05 Hradec Kralove, Czech Republic; marian.kacerovsky@fnhk.cz; 3 Department of Research and Development, University Hospital Hradec Kralove, 500 05 Hradec Kralove, Czech Republic; zdenek.zadak@fnhk.cz

**Keywords:** maternal nutrition, pregnancy outcomes, maternal diet, birth weight, birth length, protein, pregnancy, fetus, macronutrients, energy requirements

## Abstract

Maternal nutrition and metabolism play important roles for the well-being of both mother and fetus during pregnancy. This longitudinal study brings an original evaluation of the relationship between the nutritional energy and macronutrients intake (NEMI) and pregnancy outcomes and an assessment of the changes in such intake over the previous ten years. Sixty-five healthy Czech pregnant women were examined in three pregnancy periods (1st: 17th–27th; 2nd: 28th–35th; 3rd: 36th–38th gestational weeks). Results of 7-day dietary records were analyzed using NutriDan software. Energy intake decreased from 30.0 kcal/kg to 25.0 kcal/kg during pregnancy. The data also showed a decrease in macronutrients intake (*p* < 0.0001) with the advancing stage of pregnancy. Positive correlations were demonstrated between NEMI and birth weight (*r* = 0.410, *p* < 0.001). In the second pregnancy period, NEMI (excluding carbohydrates) positively associated with neonatal birth length (*p* < 0.01) and negatively with duration of birth (*p* < 0.05). An increased NEMI in the last period of pregnancy shortened the length of pregnancy.

## 1. Introduction

The intrauterine development of the fetus is one of the most vulnerable periods in human life as it depends on the intake of nutrients from the mother’s circulation [1,2]. Pregnancy is associated with increased needs for maternal energy and nutrition intake in order to meet nutritional demands of the developing fetus. Inadequate diets that lead to nutrient deficiencies as well as lack of energy intake (EI) can have a substantial impact on pregnancy outcomes and neonatal health. Energy and nutritional restriction disrupt proper fetal development and may result in disease persisting into the individual’s subsequent life, including type II diabetes, hypertension, and cardiovascular disease [3]. Therefore, understanding the relation between maternal nutrition and pregnancy outcomes may provide a basis for nutritional interventions that will improve birth outcomes and long-term quality of life [4]. In addition, knowledge of maternal nutritive status can help to optimize nutritional recommendation for pregnancy in clinical practice.

In recent years, interest has been growing about the relationship between women’s nutrition during pregnancy and birth parameters, which is positively related to perinatal survival [5]. However, results of these studies are inconsistent. The majority of the studies assess diet only at one or two time points during pregnancy. Some experimental studies confirm the effect of maternal protein intake (PI) on birth weight [5,6,7]. Additionally, an Iranian study described the significant positive correlation of EI in the end of the third trimester with neonate birth weight [6]. Sharma et al. has shown that higher consumption of carbohydrates was associated with an increase in birth weight and conversely, increasing fat intake (FI) with low birth weight [8]. Furthermore, Godfrey et al. found that increasing carbohydrate intake (CI) in early pregnancy and decreasing dairy and animal proteins in late pregnancy was associated with decreased birth weight [9]. According to GUSTO (Growing Up in Singapore Towards Healthy Outcomes) researchers, maternal macronutrient intake during pregnancy was not associated with birth weight, but lower PI correlated with longer birth length [10]. Another study interpreted positive correlation of neonatal length with EI and PI [11].

In the last ten years, there has been one longitudinal study which investigated maternal nutrition during the entire pregnancy, but not the effect on birth parameters [12]. Because the current state is unknown, the aim of this study was to evaluate nutritional and macronutrients intake (NEMI) in relation to pregnancy outcomes in Czech pregnant women and to assess the changes over the past ten years.

## 2. Materials and Methods

### 2.1. Subject Characteristics

Sixty-seven healthy Czech pregnant women with singleton gestation were enrolled into this longitudinal study. They were volunteers from prenatal courses of University Hospital in Hradec Kralove. Two of these women were excluded from the study due to thyroid pathology. The other sixty-five women were euthyroid, normoglycemic, not anemic, non-users of chronic medications, non-smokers, and non-users of alcohol or drugs. These healthy pregnant women were under the care of an obstetrician during the study. The measurements were conducted in three pregnancy periods (G1–G3) and on the day of delivery. Period G1 was from 17th to 27th gestation week (gw), when minimal maternal body changes and fetus development are recorded. Period G2 was defined as between 28th and 35th gw. This period of pregnancy is characterized by intensive fetal progress. The G3 period between 36th and 38th gw of pregnancy signifies the ending of pregnant status and time to delivery. On the day of delivery, all women were examined for anthropometric parameters.

The number of women examined varied in each period: during the G1 (*n* = 48) and G2 periods (*n* = 57) for personal and health reasons; in the G3 period (*n* = 52) due to preterm birth; and on the day of delivery due to birth in another city or because of quick labor.

Our study was performed under the Project Identification Code 201502 S06P (date of acceptance 21st January 2015) with the approval of the Ethics committee of the University Hospital in Hradec Kralove. Before each examination, all the subjects signed informed consent in accordance with the guidelines of the Declaration of Helsinki.

### 2.2. Anthropometrics

#### 2.2.1. Measurement of Pregnant Women

Anthropometric parameters were collected. A stadiometer (Mechanical Column Scale M20313) was used for measurement of height (to the nearest 0.5 cm) at the first study visit. Body weight was measured by calibrated scale (Tanita Corporation Japan), to the nearest 0.05 kg in all study periods. All measurements were performed by two trained examiners.

Maternal body mass index (BMI) was calculated from the observed height and weight. Pre-pregnancy body weight was determined from interview at the first visit.

#### 2.2.2. Birth Outcomes

The main measurements of pregnancy outcomes were the women’s weight gain (WG) during pregnancy (kg), pregnancy length (days), birth weight (kg), and birth length (cm). WG was calculated from weight on the day of delivery and the reported pre-pregnancy weight. Other parameters were obtained from measurements or hospital medical reports on the delivery.

### 2.3. Nutrition Data

Nutritional assessment was dependent on self-reported dietary intake records collected over seven consecutive days. Subjects noted the amount of consumed food and liquids during the previous week before each examination. All women were instructed in detail on how to estimate portion size. The intake of supplements was not included in the food intake. Any ambiguity and incompleteness of the dietary records were resolved in the presence of the women and the records updated. Nutritional analysis was conducted using professional software NutriDan (Danone Institut, Benesov, Czech Republic). The results of this analysis were compared with the nutritional results of the study ten years previously [12], and with the recommended dietary allowances (RDA), known as DACH doses (RDA from near Central European countries—Germany, Austria, Switzerland) [13].

### 2.4. Statistical Analysis

The data were analyzed using the programs GraphPadPrism8 (GraphPadSoftware, La Jolla, CA, USA), and Excel 2019 (Microsoft, Redmont, WA, USA). Measurements of anthropometric parameters, nutrition, and measurements on the day of delivery were assessed by descriptive statistics and expressed as median (25th percentile: 75th percentile). According to the data distribution, nonparametric Kruskal–Wallis test was used for the analysis of the differences in the obtained parameters and differences between each day of the week over the pregnancy periods. Spearman’s correlation analysis was used to describe the dependence between nutrition and neonatal parameters. For each subject, 7-day nutritional records were analyzed in all three pregnancy periods, represented by over 1000 pairs. Wilcoxon rank sum test was applied for testing differences in our obtained values from results ten years ago and RDA, too. Results were considered significant for *p* < 0.05.

## 3. Results

The baseline characteristics of the subjects and the birth parameters are shown in Table 1. The median of the women’s pre-pregnancy weight was 63.0 kg (57.0:69.0). The BMI and the weight of a pregnant woman increased with advancing pregnancy. The median of WG was 5.1 kg (3.2:7.8) in G1 period, 9.4 kg (7.1:11.8) in G2 period, 13.8 kg (10.3:15.9) in G3 period, and 14.5 kg (11.2:19.4) on the day of delivery. The pregnancy outcome characteristics expressed in medians were: length of pregnancy 283 days (275: 286), duration of birth 512 min (314:764), birth weight 3.5 kg (3.1:3.6), and birth length 49.0 cm (47.0:51.0).

With the increasing weight of the pregnant woman, there was a decrease in EI expressed in kcal per kg of body weight. The median EI was 30.0 kcal/kg, 26.9 kcal/kg, and 25.0 kcal/kg, respectively, in each pregnancy period. Nutritional energy and substrate intakes per kilogram of the woman’s weight also decreased during the individual periods of pregnancy. Nutritional changes between the three periods of pregnancy (with the exception of cholesterol, lactose, monosaccharides (MS), and disaccharides (DS)) were statistically significantly different (Table 2).

Differences in NEMI of Czech pregnant women from the previous ten years are presented in Table 3. In G2 (*p* < 0.0001) and G3 (*p* < 0.001) pregnancy periods, EI was significantly lower than ten years ago. In this current research, we found a decreasing trend in NEMI throughout the individual pregnancy periods. Ten years ago, NEMI was highest during the second trimester of pregnancy. Since then, increases in PI of about 6% (*p* < 0.0001) and 2% (*p* < 0.05) have been observed in the G1 and G3 periods, respectively. In the G1 pregnancy period, FI and polyunsaturated fatty acid (PUFA) intakes were significantly higher compared to 10 years prior. The G3 period showed 5% higher cholesterol consumption and CI decreased significantly (*p* < 0.0001) in each period, by 10% in G1, 20% in G2, and 15% in G3 during the last decade.

Table 4 shows the association of NEMI with pregnancy outcome parameters. The obtained values indicated a negative correlation of NEMI with duration of birth, especially in G2 period. PI (*p* < 0.001) and PUFA (*p* < 0.001) intake showed the greatest statistical significance in that G2 period. In addition, a significant negative association was observed between maternal nutritional intake in G1 period and the length of the pregnancy. The G3 period displayed the greatest number of negative correlations between NEMI and pregnancy length. The associations between pregnancy length and EI (*p* < 0.001), monounsaturated fatty acid (MUFA) (*p* < 0.001), and CI (*p* < 0.001) showed the strongest correlation. Assessment of the last period of pregnancy indicated a significant interrelationship between NEMI and maternal WG. Essentially, PI and fiber consumption correlated with a reduction in WG of pregnant women. In contrast, maternal NEMI was positively associated with neonatal birth weight throughout the whole pregnancy. The intakes of PUFA and cholesterol in the G2 period were the only exceptions, with no positive correlation noted. Furthermore, we observed a positive association between NEMI and birth length during the second pregnancy period. EI (*p* < 0.01), PI (*p* < 0.01), FI (*p* < 0.001) and intake of MUFA (*p* < 0.01), SFA (*p* < 0.01), and lactose (*p* < 0.05) per kilogram of the woman’s weight during the G2 period significantly correlated with birth length.

## 4. Discussion

During pregnancy, there are metabolic changes associated with both development in maternal body composition and with fetal growth. The question to be answered is whether the maternal diet affects the offspring parameters.

In the present study, we disclose a strong association of maternal diet and pregnancy outcomes, contributing to knowledge about fetal demand for an appropriate nutritional status of the mother.

Our analysis provides evidence of the influence of NEMI during all three pregnancy periods, which has not been well-described in previous studies; although some studies have investigated the association between maternal nutrition and birth parameters, they have focused on a selected period of pregnancy [6,14,15,16,17]. This longitudinal study demonstrated a decline in NEMI during gestation. Despite this decreasing trend, comparison of our nutritional analysis results with DACH recommended doses did not reveal large variations. DACH recommends a total daily calorie intake of 2355 kcal for 25 to 50-year-old pregnant women with ordinary levels of physical activity (PAL 1.6). According to DACH recommendations, our subjects received 12.5%, 16.5%, and 16.7% less energy during the first, second, and third pregnancy periods, respectively. In contrast, compliance of PI with DACH recommendations noted a higher PI than the recommended dose (58 g) during the whole pregnancy. Carbohydrates should represent more than 50% and fat 30–35% of the total diet energy intake during pregnancy. Interestingly, CI was below the recommended doses in all trimesters, while FI was slightly higher than the recommendation in the first two trimesters [13].

By comparing our results with those of a previous study involving Czech pregnant women [12], we have now demonstrated lower CI and FI over the last ten years. In contrast to the research ten years ago, an increase in PI was observed in our research subjects in the early and late terms of pregnancy. These findings reflect the change in lifestyle and living conditions over the 10 years, and support the modern trend which promotes protein consumption in the nutritional field in preference to CI. In general, the prevalence of obesity has increased over time, and therefore the question of body composition, weight control, and also healthy nutrition currently has a significant impact on the population. Interestingly, this study noted the greatest effect of nutrition on maternal WG only in the G3 pregnancy period.

Our study showed a positive relationship between EI, CI, FI, and PI and birth weight over all the periods of pregnancy. We uncovered positive significant correlations of FI and intake of its sub-components with neonatal weight, except for cholesterol and PUFA in the G2 period. In this connection, the Healthy Start Study did not find an association between maternal FI and birth weight [14]. By contrast, our observations are in line with the findings of other overseas studies [18,19,20].

Additionally, this analysis emphasizes the importance of FI not only in the early or later stages of pregnancy but also throughout the whole pregnancy. The crucial role of fatty acids in fetal and infant growth and central nervous system development has been already confirmed [18]. In this respect, a recent study found positive association between FI and neonate length [21]. To our knowledge, only a few other studies compared the effect of nutrition on the length-of-pregnancy outcome [10,11,17,22,23]. Our analysis reveals a close correlation of FI and PI with birth length and showed that higher FI, intake of its sub-components, and PI contributed to increasing birth length, principally during the G2 pregnancy period. This may be because the greater part of placental growth is completed in the second trimester, prior to the large final trimester increase in the weight of the fetus [24,25].

On the other hand, our current study is not consistent with the association of nutritional intakes during pregnancy with birth parameters recorded in some other studies. Some researchers obtained results opposite to our findings [10,17,23,24]; another did not observe any significant association between maternal macronutrient intake and pregnancy outcomes [15,22,26]. This difference may be due to their 3-day records, unlike our daily records which provide quantitatively more accurate information and confer greater validity (also due to the finding that NEMI did not differ from day to day). Other partial disparities include differences of nationality, ethnic groups, or the composition of food. Additionally, we used a slightly different assessment of nutritional intake, using kilograms per kilogram of maternal body weight, which more personalized the needs of the subjects and increased the validity of the results. This approach also minimizes any confusing effect of maternal weight status [17].

In the evaluation of maternal nutrition, our findings showed that increased EI, PI, FI, SFA, MUFA, PUFA, and PS intake in the second pregnancy period correlated with shortened duration of the birth. Of the mentioned substrates, proteins are the macronutrients that had the greatest influence on duration of birth. Interestingly, in the last phase of pregnancy, the intake of macronutrients, especially carbohydrates, was significantly related to the pregnancy length. The results showed that increased NEMI can contribute to shortening the length of pregnancy. Moreover, we found significant positive association of birth weight (*r* = 0.375, *p* < 0.0001) and birth length (*r* = 0.489, *p* < 0.0001) with pregnancy length. We suggest an explanation of this finding as being that higher nutritional intake increases the birth weight, thus influencing the maternal organism towards earlier albeit not preterm labor. It is perceived as a prevention of large birth size, which makes labor more difficult.

In the light of current knowledge, our study emphasizes that the nutrition the woman supplies to the fetus is one of the factors contributing to determination of fetal growth. The association of NEMI with pregnancy outcomes during the whole pregnancy is a novel finding. Although this study was performed with high attention to precision, more studies of a similar nature would help to contribute to a better understanding of region-specific habitual diets and birth outcomes.

## 5. Conclusions

The findings of this longitudinal study describe the changes in nutritional energy and substrate intakes of Czech pregnant women in comparison with the population ten years previously, and have demonstrated the relationship of maternal diet with pregnancy outcomes throughout all pregnancy stages.

## Figures and Tables

**Table 1:** Characteristics of pregnant women.

	G1	G2	G3	D	*p*-Value
Pregnant women (*n*)	48	57	52	52	
Maternal age (years)	29 (28:31)	29 (27:31)	29 (27:31)	29 (27:31)	0.6574
Height (cm)	166.5 (162.0:171.0)	167.0 (162.0:171.0)	167.0 (162.0:171.0)	167.0 (162.0:171.0)	0.9008
Weight (kg)	67.8 (63.0:75.1)	71.5 (66.5:77.2)	76.5 (70.5:83.7)	76.9 (70.2:86.5)	<0.0001
BMI (kg/m^2^)	24.1 (21.9:28.3)	25.7 (23.6:28.3)	27.3 (25.1:30.9)	27.7 (25.1:32.2)	0.0001

Values are presented as median (25th percentile: 75th percentile). G1—pregnancy period from 17th to 27th gestation week, G2—pregnancy period from 28th to 35th gestation week, G3—pregnancy period from 36th to 38th gestation week, D—the day of delivery, BMI—body mass index. *p*-value showed the differences in the obtained parameters over the pregnancy periods and the day of delivery (nonparametric Kruskal–Wallis test).

**Table 2:** Nutritive intake of energy and macronutrients during pregnancy.

	G1	G2	G3	*p*-Value
EI (kcal/kg)	30.0 (23.6:37.5)	26.9 (21.1:32.3)	25.0 (20.2:30.7)	<0.0001
PI (g/kg)	1.2 (0.9:1.4)	1.0 (0.8:1.3)	1.0 (0.7:1.2)	<0.0001
FI (g/kg)	1.1 (0.8:1.5)	1.0 (0.7:1.3)	0.4 (0.3:0.5)	<0.0001
SFA (g/kg)	0.4 (0.3:0.6)	0.4 (0.3:0.5)	0.3 (0.2:0.4)	0.0034
MUFA (g/kg)	0.3 (0.2:0.5)	0.3 (0.2:0.4)	0.1 (< 0.1:0.2)	0.0005
PUFA (g/kg)	0.2 (0.1:0.3)	0.2 (0.1:0.2)	4.4 (2.7:6.6)	<0.0001
Cholesterol (mg/kg)	4.3 (2.7:6.7)	4.2 (2.7:6.2)	2.9 (2.4:3.6)	0.6413
CI (g/kg)	3.5 (2.7:4.5)	3.1 (2.4:3.8)	1.0 (0.7:1.4)	<0.0001
MS and DS (g/kg)	1.1 (0.7:1.6)	1.0 (0.7:1.4)	1.6 (1.2:2.0)	0.3104
Lactose (g/kg)	0.1 (0.1:0.2)	0.1 (< 0.1:0.2)	0.1 (< 0.1:0.2)	0.1506
PS (g/kg)	2.0 (1.5:2,5)	1.7 (1.3:2.1)	1.6 (1.2:2.0)	<0.0001
Fiber (g/kg)	0.3 (0.2:0.4)	0.3 (0.2:0.3)	0.2 (0.2:0.3)	<0.0001

Values are presented as median (25th percentile: 75th percentile). G1—pregnancy period from 17th to 27th gestation week, G2—pregnancy period from 28th to 35th gestation week, G3—pregnancy period from 36th to 38th gestation week, EI—energy intake, PI—protein intake, FI—fat intake, CI—carbohydrates intake, SFA—saturated fatty acid, MUFA—monounsaturated fatty acid, PUFA—polyunsaturated fatty acid, MS—monosaccharides, DS—disaccharides, PS—polysaccharides. *p*-value showed the differences in the obtained parameters over the pregnancy periods (nonparametric Kruskal–Wallis test).

**Table 3:** The comparison of energy and macronutrients intakes with values ten years ago.

	G1			G2			G3		
	RS	10 Years Ago	δ	RS	10 Years Ago	δ	RS	10 Years Ago	δ
EI (kcal)	2061 (1693:2547)	2074 ± 413.1	99.37	1965 (1623:2315)	2251 ± 479.0	87.29 ^****^	1962 (1616:2275)	2069 ± 364.6	94.83 ^***^
PI (g)	79.91 (65.78:100.6)	75.7 ± 15.6	105.56 ^****^	75.63 (59.22:92.09)	80.6 ± 16.7	93.83 ^***^	73.94 (58.06:92.26)	72.3 ± 11.2	102.27 ^*^
FI (g)	76.66 (57.53:103.1)	72.9 ± 16.5	105.16 ^***^	75.06 (54.64:93.03)	83.7 ± 31.0	89.68 ^****^	72.4 (54.47:92.07)	74.3 ± 15.8	97.44
SFA (g)	30.42 (22.13:40.39)	30.1 ± 7.3	101.06	29.96 (21.34:38.45)	33.8 ± 9.9	88.64 ^****^	28.85 (21.76:39.56)	30.6 ± 7.5	94.28
MUFA (g)	22.71 (16.75:31.3)	21.6 ± 5.3 ^***^	105.14	22.57 (16.6:29.62)	25.6 ± 11.0	88.16 ^****^	22.46 (16.61:28.23)	22.6 ± 4.9	99.38
PUFA (g)	13.01 (8.93:20.37)	12.8 ± 3.9	101.64 ^***^	11.84 (8.43:16.51)	15.1 ± 9.0	78.41 ^****^	11.07 (7.27:16.33)	12.8 ± 3.7	86.48 ^**^
Cholesterol (mg)	292 (191.3:472.5)	313.5 ± 105.4	93.14	304.1 (204.5:475)	351.5 ± 102.1	86.51	345.3 (216.6:516)	328.6 ± 85.3	105.08 ^**^
CI (g)	239.6 (189.9:297.1)	267.3 ± 64.2	89.64 ^****^	223.3 (182.4:274.4)	280.9 ± 58.6	79.49 ^****^	225.2 (184:276.2)	266.2 ± 55.6	84.60 ^****^
Fiber (g)	21.57 (16.24:27.85)	22.6 ± 5.3	95.44	19.7 (15.74:24.52)	23.7 ± 6.3	83.12 ^****^	18.98 (14.24:24.94)	22.3 ± 5.1	85.11 ^****^

Absolute values G1–G3 given as median (25th percentile: 75th percentile). Absolute values 10 years ago given as the mean ± standard deviation. Wilcoxon rank sum test was used for testing the significance of differences between recent study and the results 10 years ago. * *p* < 0.05, ** *p* < 0.01, *** *p* < 0.001, **** *p* < 0.0001. **δ**—differences between recent study and values 10 years ago, RS—recent study, G1—pregnancy period from 17th to 27th gestation week, G2—pregnancy period from 28th to 35th gestation week, G3—pregnancy period from 36th to 38th gestation week, EI—energy intake, PI—protein intake, FI—fat intake, CI—carbohydrates intake, SFA—saturated fatty acid, MUFA—monounsaturated fatty acid, PUFA—polyunsaturated fatty acid, MS—monosaccharides, DS—disaccharides, PS—polysaccharides.

**Table 4:** Correlation analysis of energy and macronutrients intake and parameters of pregnancy outcomes.

		WG (kg)	Pregnancy Length (days)	Duration of Birth (min)	Birth Weight (kg/kg)	Birth Length (cm/cm)
G1	EI (kcal/kg)	−0.008	−0.133 ^*^	−0.025	0.405 ^***^	0.059
	PI (g/kg)	−0.089	−0.101	−0.077	0.322 ^***^	0.084
	FI (g/kg)	−0.016	−0.098	−0.079	0.410 ^***^	0.094
	SFA (g/kg)	0.017	−0.127	−0.027	0.392 ^***^	0.073
	MUFA (g/kg)	−0.019	−0.074	−0.050	0.356 ^***^	0.111
	PUFA (g/kg)	−0.062	−0.053	−0.104	0.304 ^***^	0.043
	Cholesterol (mg/kg)	0.099	0.050	0.018	0.277 ^***^	0.046
	CI (g/kg)	0.032	−0.131 ^*^	0.047	0.309 ^***^	0.013
	MS and DS (g/kg)	−0.084	−0.101	0.011	0.264 ^***^	0.037
	Lactose (g/kg)	−0.066	−0.100	0.046	0.236 ^***^	−0.062
	PS (g/kg)	0.098	−0.128	0.076	0.187 ^**^	−0.032
	Fiber (g/kg)	−0.016	−0.064	0.012	0.319 ^***^	0.056
G2	EI (kcal/kg)	0.039	−0.032	−0.162 ^**^	0.246 ^***^	0.149 ^**^
	PI (g/kg)	−0.004	−0.025	−0.259 ^***^	0.158 ^***^	0.136 ^**^
	FI (g/kg)	0.041	−0.012	−0.165 ^**^	0.209 ^***^	0.158 ^***^
	SFA (g/kg)	0.073	−0.003	−0.124 ^*^	0.211 ^***^	0.159 ^**^
	MUFA (g/kg)	0.008	0.007	−0.146 ^*^	0.198 ^***^	0.157 ^**^
	PUFA (g/kg)	−0.042	0.029	−0.201 ^***^	0.059	0.036
	Cholesterol (mg/kg)	0.051	0.025	−0.063	0.108	0.029
	CI (g/kg)	0.054	−0.027	0.058	0.235 ^***^	0.086
	MS and DS (g/kg)	0.078	−0.009	−0.038	0.129 ^*^	0.100
	Lactose (g/kg)	0.038	0.115 ^*^	0.010	0.152 ^*^	0.164 ^*^
	PS (g/kg)	0.002	−0.016	−0.050	0.196 ^***^	0.027
	Fiber (g/kg)	−0.043	−0.087	−0.127 ^*^	0.162 ^**^	0.051
G3	EI (kcal/kg)	−0.137 ^**^	−0.199 ^***^	−0.078	0.245 ^***^	0.085
	PI (g/kg)	−0.260 ^****^	−0.147 ^**^	−0.103	0.240 ^***^	0.135 ^*^
	FI (g/kg)	0.109 ^*^	−0.152 ^**^	−0.034	0.140 ^***^	0.078
	SFA (g/kg)	−0.077	−0.146 ^**^	−0.079	0.157 ^**^	0.098
	MUFA (g/kg)	−0.130 ^*^	−0.177 ^***^	−0.019	0.139 ^*^	0.026
	PUFA (g/kg)	−0.095	−0.049	0.016	0.080	0.076
	Cholesterol (mg/kg)	−0.146 ^**^	−0.127 ^*^	−0.019	0.110 ^*^	−0.058
	CI (g/kg)	−0.099	−0.192 ^***^	−0.105	0.253 ^***^	0.047
	MS and DS (g/kg)	0.009	−0.166 ^**^	−0.068	0.165 ^*^	0.019
	Lactose (g/kg)	−0.114 ^*^	−0.004	−0.029	0.130 ^*^	0.136 ^*^
	PS (g/kg)	−0.171 ^**^	−0.120 ^*^	−0.118 ^*^	0.186 ^***^	0.038
	Fiber (g/kg)	−0.262 ^***^	−0.156	−0.109	0.228 ^***^	0.053

Values given as *r*-value. Spearman’s correlation analysis was used to testing the dependence between nutrition and neonatal parameters. * *p* < 0.05, ** *p* < 0.01, *** *p* < 0.001, **** *p* < 0.0001. G1—pregnancy period from 17th to 27th gestation week, G2—pregnancy period from 28th to 35th gestation week, G3—pregnancy period from 36th to 38th gestation week, WG—weight gain, EI—energy intake, PI—protein intake, FI—fat intake, CI—carbohydrates intake, SFA—saturated fat acid, MUFA—monounsaturated fatty acid, PUFA—polyunsaturated fatty acid, MS—monosaccharides, DS—disaccharides, PS—polysaccharides.

## Abbreviations

NEMI	nutritional energy and macronutrients intake
EI	energy intake
PI	protein intake
CI	carbohydrates intake
FI	fat intake
gw	gestational week
WG	weight gain
BMI	body mass index
SFA	saturated fatty acid
MUFA	monounsaturated fatty acid
PUFA	polyunsaturated fatty acid
MS	monosaccharides
DS	disaccharides
PS	polysaccharides
RDA	recommended dietary allowances

## References

[1] Muthayya S. (2009). Maternal nutrition & low birth weight—What is really important?. Indian J. Med. Res..

[2] Ozanne S.E., Nicholas Hales C. (2005). Poor fetal growth followed by rapid postnatal catch-up growth leads to premature death. Mech. Ageing Dev..

[3] Myles M., Gennaro S., Dubois N., O’Connor C., Roberts K. (2017). Nutrition of Black Women during Pregnancy. J. Obstet. Gynecol. Neonatal Nurs..

[4] Abu-Saad K., Fraser D. (2010). Maternal nutrition and birth outcomes. Epidemiol. Rev..

[5] Cuco G., Arija V., Iranzo R., Vila J., Prieto M.T., Fernandez-Ballart J. (2006). Association of maternal protein intake before conception and throughout pregnancy with birth weight. Acta Obstet. Gynecol. Scand..

[6] Khoushabi F., Saraswathi G. (2010). Impact of nutritional status on birth weight of neonates in Zahedan City, Iran. Nutr. Res. Pract..

[7] Moore V.M., Davies M.J., Willson K.J., Worsley A., Robinson J.S. (2004). Dietary composition of pregnant women is related to size of the baby at birth. J. Nutr..

[8] Sharma S.S., Greenwood D.C., Simpson N.A.B., Cade J.E. (2018). Is dietary macronutrient composition during pregnancy associated with offspring birth weight? An observational study. Br. J. Nutr..

[9] Godfrey K., Robinson S., Barker D.J., Osmond C., Cox V. (1996). Maternal nutrition in early and late pregnancy in relation to placental and fetal growth. BMJ.

[10] Chong M.F., Chia A.R., Colega M., Tint M.T., Aris I.M., Chong Y.S., Gluckman P., Godfrey K.M., Kwek K., Saw S.M. (2015). Maternal Protein Intake during Pregnancy Is Not Associated with Offspring Birth Weight in a Multiethnic Asian Population. J. Nutr..

[11] Gala U.M., Godhia M.L., Nandanwar Y.S. (2016). Effect of Maternal Nutritional Status on Birth Outcome. J. Adv. Nutr. Health Sci..

[12] Hronek M., Doubkova P., Hrnciarikova D., Zadak Z. (2013). Dietary intake of energy and nutrients in relation to resting energy expenditure and anthropometric parameters of Czech pregnant women. Eur. J. Nutr..

[13] Stranska K., Andelova M., Stransky M., Kohout P. (2011). Referenční Hodnoty Pro Příjem Živin.

[14] Crume T.L., Brinton J.T., Shapiro A., Kaar J., Glueck D.H., Siega-Riz A.M., Dabelea D. (2016). Maternal dietary intake during pregnancy and offspring body composition: The Healthy Start Study. Am. J. Obstet. Gynecol..

[15] Lagiou P., Tamimi R.M., Mucci L.A., Adami H.O., Hsieh C.C., Trichopoulos D. (2004). Diet during pregnancy in relation to maternal weight gain and birth size. Eur. J. Clin. Nutr..

[16] Pathirathna M.L., Sekijima K., Sadakata M., Fujiwara N., Muramatsu Y., Wimalasiri K.M.S. (2017). Impact of Second Trimester Maternal Dietary Intake on Gestational Weight Gain and Neonatal Birth Weight. Nutrients.

[17] Switkowski K.M., Jacques P.F., Must A., Kleinman K.P., Gillman M.W., Oken E. (2016). Maternal protein intake during pregnancy and linear growth in the offspring. Am. J. Clin. Nutr..

[18] Bonakdar S.A., Dorosty Motlagh A.R., Bagherniya M., Ranjbar G., Daryabeygi-Khotbehsara R., Mohajeri S.A.R., Safarian M. (2019). Pre-pregnancy Body Mass Index and Maternal Nutrition in Relation to Infant Birth Size. Clin. Nutr. Res..

[19] Mani I., Dwarkanath P., Thomas T., Thomas A., Kurpad A.V. (2016). Maternal fat and fatty acid intake and birth outcomes in a South Indian population. Int. J. Epidemiol..

[20] Salvig J.D., Lamont R.F. (2011). Evidence regarding an effect of marine n-3 fatty acids on preterm birth: A systematic review and meta-analysis. Acta Obstet. Gynecol. Scand..

[21] Hjertholm K.G., Iversen P.O., Holmboe-Ottesen G., Mdala I., Munthali A., Maleta K., Shi Z., Ferguson E., Kamudoni P. (2018). Maternal dietary intake during pregnancy and its association to birth size in rural Malawi: A cross-sectional study. Matern. Child Nutr..

[22] Andreasyan K., Ponsonby A.L., Dwyer T., Morley R., Riley M., Dear K., Cochrane J. (2007). Higher maternal dietary protein intake in late pregnancy is associated with a lower infant ponderal index at birth. Eur. J. Clin. Nutr..

[23] Grandy M., Snowden J.M., Boone-Heinonen J., Purnell J.Q., Thornburg K.L., Marshall N.E. (2018). Poorer maternal diet quality and increased birth weight. J. Matern. -Fetal Neonatal Med..

[24] Jung Y.M., Choi M.J. (2017). Nutrient Intake according to Weight Gain during Pregnancy, Job Status, and Household Income. Clin. Nutr. Res..

[25] Salafia C.M., Charles A.K., Maas E.M. (2006). Placenta and fetal growth restriction. Clin. Obstet. Gynecol..

[26] Kubota K., Itoh H., Tasaka M., Naito H., Fukuoka Y., Muramatsu Kato K., Kohmura Y.K., Sugihara K., Kanayama N., Hamamatsu Birth Cohort Study Team (2013). Changes of maternal dietary intake, bodyweight and fetal growth throughout pregnancy in pregnant Japanese women. J. Obstet. Gynaecol. Res..

